# Supplementary material for: Chromothripsis during telomere crisis is independent of NHEJ, and consistent with a replicative origin
Source: Genome Res. 2019 May;29(5):737–49. doi: 10.1101/gr.240705.118 (PMC6499312; doi:10.1101/gr.240705.118)
Supplement: Supplemental Material [file supp_gr.240705.118_Supplemental_file_1.zip › contigs/annotated_contigs/DB40/contig.2.DB40_length_761_mean_cov_8.97897503285.docx]

**DB40_length_761_mean_cov_8.97897503285**

```
GAAATGGTTATGGTAATTACAGTAAAACTATTCAACATAGTGTTCTTT|AAGCTGAAACTGGATCCCTTCCTTACACCTTATACAAAAA
 >chr4:66998594-66998724 + E=2e-66 p=0e+00       >chr9:5036665-5037378 + E=0e+00
TTAATTCAACAGGGATTAAAGACTTAAATGTTAGACCTAAAA|CCATAAAAACCCTAGAAGAAAACCTAGGCAATACCATTCAGGACAT

AAGCATGGGCAAGGACTTCATGACTAAAACACCAAAAGCAATGGCAACAAAAGCCAGACTTGACAAATGGGATCTCATTAAACCAAAGA

GCTTCTGCACAGCAAAAGAAACTACCATCAGAGTGAACAGGCAACCTACAGAATGGGAGAACATTTTTGTAATCTACTCATCTGATAAA

GGGCTAATATCCAGAATATACAATGAACTCTAACAAATTTACAAGAAAACAACAACCCCATCAAAAAGTGGGCGAAGGATACGAACAGA

CACTTCTTGAAAGAAGACATTTATGCAGCCAAAAGACATGAAAAAATGCTCATCACCACTGGCCATCAGAGAAATGCAAATCAAAACCA

CAATGAGATACCATCTCACACCAGTTAGAATGGCGATCATTAAAAAGTCAGGAAACAACAGGTGCTGGAGAGAATGTGGAGAAACAGGA

ACACCTTTACACTGTTGGTGGGACAGTAAACTAGTTCAACCCATGTGGAAGACAGTGTGGTGATTCCTCAGGGATCTAGAACTAGAAAT

ACCATTTGACCCAGCCATCCCATTACTGGGTATATACCCAAAGGATTATAA
```
